# Anchoring effects in the assessment of papers: An empirical survey of citing authors

**DOI:** 10.1371/journal.pone.0283893

**Published:** 2023-03-31

**Authors:** Lutz Bornmann, Christian Ganser, Alexander Tekles

**Affiliations:** 1 Science Policy and Strategy Department, Administrative Headquarters of the Max Planck Society, Munich, Germany; 2 Department of Sociology, Ludwig-Maximilians-Universität Munich, Munich, Germany; Iuliu Hațieganu University of Medicine and Pharmacy: Universitatea de Medicina si Farmacie Iuliu Hatieganu, ROMANIA

## Abstract

In our study, we have empirically studied the assessment of cited papers within the framework of the anchoring-and-adjustment heuristic. We are interested in the question whether the assessment of a paper can be influenced by numerical information that act as an anchor (e.g. citation impact). We have undertaken a survey of corresponding authors with an available email address in the Web of Science database. The authors were asked to assess the quality of papers that they cited in previous papers. Some authors were assigned to three treatment groups that receive further information alongside the cited paper: citation impact information, information on the publishing journal (journal impact factor) or a numerical access code to enter the survey. The control group did not receive any further numerical information. We are interested in whether possible adjustments in the assessments can not only be produced by quality-related information (citation impact or journal impact), but also by numbers that are not related to quality, i.e. the access code. Our results show that the quality assessments of papers seem to depend on the citation impact information of single papers. The other information (anchors) such as an arbitrary number (an access code) and journal impact information did not play a (important) role in the assessments of papers. The results point to a possible anchoring bias caused by insufficient adjustment: it seems that the respondents assessed cited papers in another way when they observed paper impact values in the survey. We conclude that initiatives aiming at reducing the use of journal impact information in research evaluation either were already successful or overestimated the influence of this information.

## Introduction

Hammarfelt, Rushforth [1] analyzed peer review reports and identified criteria and strategies that reviewers used to decide on candidates for professorships in Sweden. It is no surprise to read in the paper that bibliometric indicators (especially the popular *h* index) play a prominent role in the assessments of the candidates. The reason for their use is understandable: since no researcher is an expert in all fields within a discipline, it appears reasonable to resort to metrics in cases of fields that are remote from those that are familiar. The use of metrics in situations with great judgment uncertainties may reach its limits, however. Let us suppose a situation in which several candidates for a professorship position are compared. It is the last round of a selection process and the candidates are very similar in terms of their performance. Their previous research papers, funds received, and teaching experience have been discussed in great detail. When it comes to the decision regarding the candidates, the summaries for all candidates are provided to the reviewers with the total citation counts. The citation counts are very different, since the candidates are from different fields within economy [see 2].

In thinking about the candidates, one of the reviewers is impressed by the very high citation counts of one candidate (someone from the field of financial economics) and observes that the other candidates are not on the same level. If the reviewer recommends this candidate for the professorship position, the reason for this decision may be the so-called ‘anchoring effect’. Tversky and Kahneman [3] introduced the anchoring-and-adjustment heuristic: “the heuristic maintains that anchoring bias is caused by insufficient adjustment because final judgements are assimilated toward the starting point of a judge’s deliberations … the anchoring effect is the disproportionate influence on decision makers to make judgments that are biased toward an initially presented value” [4]. Research on anchoring effects has shown for various assessment contexts that presented values can have a substantial effect on judgments. Values can even serve as anchors that are not directly related to the assessed objects. Thus, in the example above, it is not only citation counts that could act as an anchor, but also numerical identifiers for the candidates (or any other arbitrary number) presented with the summaries.

In this paper, we present the results of a study that has investigated various anchoring effects in the assessment of scientific work. To the best of our knowledge, this is the first time that anchoring effects are being investigated in the assessment of scientific work. We found only one study [5] in the area of research evaluation that investigated dependencies of institutional reputation assessments in university rankings on previous rankings within the anchoring framework. The investigation of anchoring effects in research evaluation is an important issue, however. Scientific progress is a key factor for the growing affluence of a society. According to Mormina [6], “the ability to generate scientific and technological knowledge (S&T) and translate it into new products or processes is a key instrument of economic growth and development” [p. 672, see also 7]. A prerequisite for scientific progress is research at the highest possible quality level. Since ‘research quality’ is a normative, ascribing concept [8,9], it depends on assessments by peers (experts in a field). To avoid false quality attributions, the assessments should be unbiased. It would inhibit scientific progress if bias factors such as anchoring effects were to significantly affect the assessment.

The design of our study is oriented towards the study by Teplitskiy, Duede [10]. The authors conducted a web-based survey by investigating the referencing behavior of citing authors. Their study had an experimental design, since the authors worked with treatment and control groups and random assignments of respondents to the groups. The respondents assigned to the groups were asked to assess the quality of references that they have cited in publications some years ago. The treatment group differs from the control group in that it received information on the (true) citation impact of the cited references. The results of the study show that the treatment harms the quality assessments of the cited references.

In our study, we have empirically studied the assessment of cited references within the framework of the anchoring-and-adjustment heuristic. Teplitskiy, Duede [10] did not conduct their study within this framework, although the effect of the treatment may be interpreted as an anchoring effect. We have investigated whether possible adjustments in the assessments of cited references can not only be produced by citation impact information, but also by figures that are not related to quality: are the adjustments specific for citations, or can they be triggered by other figures? An answer on this question may have important implications for quality assessments of papers by researchers and the role of citations in the assessment processes. Quality assessments should be based on research itself, and not on (less relevant) figures. If the assessments are dependent on these figures, their validity may be questioned.

Before we explain our research design and results in more detail below, we present a literature overview of the anchoring-and-adjustment framework (in psychology). This overview is mainly based on two comprehensive overviews published some years ago: Mussweiler, Englich [11]–and the revised chapter published by Bahnik, Englich [12]–and Furnham and Boo [4]. A brief overview of the anchoring effect research can also be found in Kahneman [13].

## Literature overview

Many decisions in research evaluation are judgments that are subject to uncertainty. Although the evaluation tasks are frequently complex and based on ambiguous information, the use of (simple) bibliometric indicators is very popular. Bornmann and Marewski [14] proposed the study of the evaluative use of bibliometrics within the fast-and-frugal heuristics research program. Well selected and informed decision strategies that are based on bibliometric indicators may serve as heuristics (rules of thumb) in the assessment of scientific work. Bornmann and Marewski [14] argue that such bibliometrics-based heuristics (BBHs) may lead to decisions in certain research evaluation situations that are at least similarly good as expensive peer review processes (that may or may not be informed by indicators). This argument is based on the results of numerous empirical studies (in psychology) that have demonstrated the comparable good performance of heuristics compared with other, more complex approaches using full information in various decision contexts.

From the mid-1970s onwards, research on decision making also revealed another side of the heuristics coin. This research is mainly based on the pioneering work by Tversky and Kahneman [3]. Their study demonstrated that the use of heuristics may lead to biases in decision making processes. The respondents in the study were asked to estimate the percentage of African nations in the United Nations (UN). An arbitrary number presumably produced by a wheel of fortune was used as a possible anchor in the estimation process: the respondents were requested to estimate whether the percentage is higher or lower than the anchor. A 20 percentage-point difference in the mean estimations (of the subsequent absolute anchoring question) revealed the strong effect of the anchor that was randomly induced. Thus, the results indicate that the estimates were (strongly) adjusted by the respondents to the anchor value.

One might think that the results by Tversky and Kahneman [3] are restricted to judgments without important implications. The results by Englich and Mussweiler [15] revealed that this is not the case, however: experienced trial judges were influenced by sentencing demands, and this influence could also be observed in situations in which the demands came from non-experts. Since the appearance of the study by Tversky and Kahneman [3], many studies have investigated anchoring effects in very different contexts. Descriptive overviews of these studies in tabular form can be found in Furnham and Boo [4]. The literature overviews of Mussweiler, Englich [11] and Furnham and Boo [4] came to very similar conclusions in their overall assessments of the prevalence of anchoring effects [see also 12]: “anchoring effects are among the most robust and easily replicated findings in psychology” [11]. Furnham and Boo [4] concluded as follows: “research in the field demonstrates that anchoring is a pervasive and robust effect in human decisions” (p. 41). Previous research even revealed that anchoring effects appeared in situations in which explicit instructions exist to correct their effect.

The robustness of the anchoring effect can be assumed, since the effect seems to exist independently of many moderating variables. Anchoring effects have been shown in comparative judgment tasks (e.g. is something larger or smaller) and in absolute judgment tasks (e.g. how high, long, or great is something) [11]. The effect seems to be independent of the extremity of the anchor (although it should not be implausibly extreme). Anchors may be numeric values or certain stimuli. Decision tasks in anchoring effect studies were trivial (e.g. an estimate of the percentage of African nations in the UN), or apocalyptic (e.g. the estimated likelihood of a nuclear war). Robust anchoring effects could be observed not only in laboratory settings such as those used by Tversky and Kahneman [3], but also in ‘real-world’ settings such as the legal judgment settings in Englich and Mussweiler [15]. Another typical ‘real-world’ setting in anchoring effect studies is consumer behavior: consumer behavior appears to be influenced by price proposals that serve as anchors.

Studies have differentiated whether or not the anchor should be relevant for the judgmental task in order to be effective. In our study, for example, citations may be interpreted as relevant anchors for the assessment of papers, but arbitrary identifiers of papers can be interpreted as being irrelevant for quality assessments. Although one might expect that only relevant anchors are effective, Furnham and Boo [4] concluded–based on the literature–that “irrelevant anchors produce similar effects in judgmental decisions in comparison to those of informational relevance anchors” (p. 38). Research also revealed that anchoring effects are observable in situations in which participants tried to work against the anchors’ influence or received a prize for presenting the best estimate.

Anchoring is usually used as a descriptive concept by pointing to an assimilation or contrast [16]. Some studies have provided possible mechanisms for explaining the observed anchoring effects in the various contexts. The proposed mechanisms have been summarized by Mussweiler, Englich [11] as follows: “At least three mechanisms may influence the initial stage of standard selection. First, a particular value may be selected as an anchor because conversational inferences suggest it as relevant. If a particular anchor is explicitly mentioned by the experimenter, then judges may well use it to subsequently compare it to the target. Second, a value may be selected as an anchor because it is easily accessible and comes to mind during the evaluation of the target. Finally, an anchor may be self-generated via an insufficient adjustment process. Judges who are provided with an implausible anchor, for example, may use this value as a starting point to generate a more plausible value, which is then compared to the target. This suggests that the alternative mechanisms of conversational inference, numeric priming, and insufficient adjustment may contribute to the selection of an anchor value” (pp. 195–196).

## Methods and data

### Study design

The numerous studies on anchoring effects show that the assimilation of judgments occurs in many natural settings (e.g. pricing or legal judgments) and thus has a great practical significance in judgments by experts and non-experts. According to Englich and Mussweiler [15], “anchoring is a pervasive and robust effect in human judgment that reliably influences numeric estimates in a variety of natural settings” (p. 1537). In this study, we have investigated anchoring effects in the assessment of scientific work. We conducted a survey of corresponding authors with an available email address in the Web of Science database (a multi-disciplinary literature database including citations). The survey was similarly designed as the survey conducted by Teplitskiy, Duede [10]. The questionnaire used by the authors can be found in their supplementary information.

Besides presenting a random anchor, there are minor differences between the questionnaire used by Teplitskiy, Duede [10] and our questionnaire.

- We added two response categories to the question “Do you remember this reference?” because of feedback we received in a pretest of our survey. Besides the original categories “Yes, I added this reference”, “Yes, a co-author added this reference” and “No, continue to next reference”, we included “Yes, we included this reference together” and “Yes, but I cannot remember who added it”. The category “No, continue to next reference” was rephrased as “No, skip this reference” because in our sample not all authors were provided with more than one reference.
- We skipped the response category “Only minor influence” for the question “Which aspects of your paper did this reference influence?” because the strength of the influence is addressed in a separate question.
- At the end of the questionnaire, we asked the respondents whether or not they had read Teplitskiy, Duede [10] or the registered report protocol [17] for this study prior to taking the survey. This allows us to control for potential bias induced by knowing the design and goal of our study.
- The design of the questionnaire differs due to using a different survey software.

The Research Ethics Committee of Social Sciences at the Ludwig-Maximilians-University (LMU) Munich has reviewed our planned survey regarding ethical aspects of the proposed research. The committee came to the following conclusion: there are no objections to the implementation of the research project. Our questionnaire and the dataset are available online [18].

The corresponding authors in our sample (with an available email address in the Web of Science database) received an email with a link to a web-based questionnaire. The authors were asked for the assessment of a paper (reference) that he/she cited in a previous publication. The authors were randomly assigned to different experimental settings in which they received (or not) (1) a randomly generated anchor that is not related to the quality of publications, (2) information about the (true) citation impact of the paper (as an anchor with possibly relevant information), or (3) information about the (true) citation impact of the journal in which the paper was published (as an anchor with possibly relevant information).

Teplitskiy, Duede [10] used the following cover letter in the email of their survey to the authors: “The *Laboratory for Innovation Science at Harvard* would like to invite you to take part in a quick (5–10 minute) survey about how researchers reference existing work when writing papers. Although citations and related metrics like the *h-index* are widely used in academia to evaluate research and allocate resources, the referencing decisions on which they are based are poorly understood. Consequently, we have selected your paper … and want to ask you about two specific references within it. Your input will help us and the broader scientific community assess the validity and limitations of existing ways of evaluating and ranking scientific works and possibly develop superior alternatives”. We used almost the same email. However, the paper selected for the survey was not named in the email because for some authors we selected more than one paper. This facilitated us to provide a substitute when a respondent could not remember a reference. Furthermore, we added the sentence “We selected you because you published an article in the years 2018–2019 in which you referenced a paper that we plan to include in our analyses” due to feedback in the pretest. This sentence was intended to explain our invitation to take part in the survey.

When the respondents decided to take part and started the survey, they were asked whether they remembered the presented references (or not). Additional citation impact information or the randomly generated anchor were presented, if the respondents have been assigned to the corresponding experimental group. Respondents assigned to the control group received none of this information. Further questions referred to how well the authors knew the paper (‘extremely well’ through to ‘not well’), how much the reference influenced the research choices in the citing paper, and which aspects of the paper were influenced by the cited references. In the final part of the questionnaire, the respondents rated the reference against possible others in the field concerning several characteristics (quality, novelty, significance, validity, generalizability, and canonical or prominent reference, respectively; the aspects canonical and prominent were randomized so that half of the respondents saw one term or the other). These are typical characteristics of quality that can be also found in other studies [19].

Further deviations from the survey design by Teplitskiy, Duede [10] were outlined in our registered report protocol [17] and are presented in the following:

1. Presentation of a journal metric: Teplitskiy, Duede [10] provided citation counts of the cited publications and its citation percentile in the questionnaire. The citation percentile is the percentage of papers in a subject category (and publication year) that received (equal to or) fewer citations than the focal paper [20]. In our questionnaire, we presented not only information on the citation percentile of single papers, but also the citation impact of the journal in which the cited reference has been published. The information on the journal impact was also presented as percentile information: the percentage of journals in a subject category (and publication year) that received (equal to or) fewer citations than the focal journal in terms of the popular journal impact factor. The impact of the journal may also serve as anchor for the respondents in assessing the cited reference. The study by Waltman and Traag [21] indicates that the value of a paper may be interpreted within two perspectives: the number of received citations and the reputation of the publishing journal. In our study, we are interested in whether the two perspectives lead to different anchoring effects in the assessments of the cited references.
2. Randomly generated anchor with irrelevant information: The citation information provided in the survey by Teplitskiy, Duede [10] may be interpreted as an anchor providing relevant information for the assessment of the quality of the cited references. We considered another anchor with irrelevant numerical information [see 22]. This second anchor was presented as an access code in the cover letter (see above). The access code was randomly generated from numbers between 1 and 99. We focused on this range to be in the same range as citation and journal percentiles. The respondents were asked for typing the code in the web-based questionnaire [see here 23]. Respondents who did not receive the anchor stimulus did not have to enter a code. In our registered report protocol we originally planned to provide letters as a code. We decided not to implement this, since we expected that letters could have an unexpected influence on the respondents.
3. Assessment of only one cited reference: Respondents in the study by Teplitskiy, Duede [10] were requested to assess more than one cited reference. We deviated from this approach: each corresponding author was asked to assess only one cited reference. The presentation of more than one cited reference may lead to undesirable dependencies in the assessment of cited references. Another advantage is the reduction in time for working on the web-based questionnaire. However, up to three substitute references were provided if a respondent could not remember a reference.
4. Assessment of the same cited reference by many respondents: We considered only papers with references in our study that have been cited by more than one citing corresponding author in our dataset. Each cited reference was assessed under three treatment group conditions (citation, journal, and access code) and the control group conditions. This should have enabled us to control the effect of the cited reference in the statistical analyses (by using models for repeated measures). However, due to the low response rate in this study, this was not possible (see below).

The bibliometric data that we used in our study originate from an in-house database developed and maintained by the Max Planck Digital Library (MPDL, Munich) and derived from the Science Citation Index Expanded (SCI-E), Social Sciences Citation Index (SSCI), Arts and Humanities Citation Index (AHCI) prepared by Clarivate (https://clarivate.com). We focused on papers published in 2018 or 2019, since we anticipated better memory performance of the citing authors for recent papers than for papers published many years ago. In the database, there are 5,608,611 papers published in 2018 or 2019. Of these papers, we considered only articles, since different types of documents may lead to different selection decisions of cited references by citing authors at that time. For reviews, cited references are selected (and read) to be included in an overview of research; for articles, cited references are more likely to be the primary starting point for own research.

In our study, we considered articles only if they have a single email address for the corresponding author in the database (some papers in the database have more than one email address). In order to identify the corresponding authors to be contacted, we included only papers that have at least one article as a linked cited reference in our database. For a linked cited reference, a corresponding source document exists in the Web of Science database. Further metadata, such as paper title, citation counts, and journal impact factor only exist for linked cited references. Furthermore, we only included those papers with a similar number of cited references, in order to control a possible effect of the number of cited references on how well the citing authors can remember a particular cited reference. For this purpose, we calculated the mode number of cited references based on the papers remaining after the aforementioned restrictions (which is 30 in our dataset). We only kept those papers from this set whose number of cited references does not differ from the mode number of cited references by more than 2.

For each of these papers, we generated the pairs of the email address of the corresponding author and the linked cited references (with the ‘article’ document type) of the papers. Only cited references of document type ‘article’ and for which the necessary metadata (paper title, abstract, DOI, citation counts, journal impact factor) are available were used for this matching procedure. From this set, we excluded all pairs for which both papers share at least one author name in order to exclude self-citations. For each paper occurring as a cited reference in the resulting pairs, we selected at least 4 email addresses, while at the same time, each email address has been selected not more than once. Note that several citing papers may have the same email address. In such cases, a maximum of 1 cited reference has been selected for an email address as well (instead of selecting 1 cited reference for each citing paper).

We used a greedy algorithm [i.e. an algorithm using a heuristic to find a locally optimal choice at each stage of the algorithm; see 24] that aims to select as many pairs of email addresses and cited references that fulfil the aforementioned conditions as possible. In a first step, this algorithm iterates over all cited references in the data in the order of the number of citing authors (first all references that have been cited by four authors, then all references that have been cited by five authors etc.). Each cited reference is matched with all citing authors that have not already been selected for other references, as long as there are at least four of such citing authors. This ensures that each reference can be assessed at least four times (i.e. under each treatment and the control condition). In a second step, up to three substitute cited references are selected for the citing authors that could be matched in the first step. The substitute cited references are matched in the order of the number of times a reference has been matched in the first step, starting with references that could only be matched four times.

This approach increases the chance to get further assessments for references that occur less often as default references, i.e. to have more cited references that are assessed at all. The process resulted in 77,872 email addresses and 16,575 cited references (without substitute references; another 21,633 papers are included as substitute references in our data). Each of the 77,872 corresponding authors has been assigned randomly to one of the three treatment groups or the control group. Each treatment group received one of the three items of information: paper impact (citation percentile for the cited article), journal impact (journal impact factor percentile for the cited article) or access code. The authors in the control group did not receive any of this information. This resulted in 19,568 authors for each group. In our study, we used the email addresses only for inviting the respondents. After collecting the data, we deleted the email addresses and analyzed the anonymized data which do not allow the identification of respondents.

In our sample, we have corresponding authors with different levels of experience in their field. We assume that the assessments of cited references are dependent on this level: senior researchers may be in a better position to assess the quality of a cited reference than junior researchers. There may be the risk, therefore, that junior researchers have been more open to biases in their assessments. In our study, we target these differences between the corresponding authors by means of their random assignments to the treatment and control groups. Furthermore, the literature overview by Furnham and Boo [4] shows that “expertise does not significantly reduce the assimilative bias in decisions that affect inexperienced laypeople” (p. 39).

### Number of respondents and ratings

After checking for structural validity, i.e. exclusion of technically invalid addresses like name@domain (without top level domain), our sample consisted of 77,872 email addresses to which we sent the invitation between February, 28^th^ and March, 3^rd^, 2022. A huge number of addresses turned out to be invalid because the addressees were unknown. The online survey tool we used (Unipark, which is based on EFS Survey by Tivian, see www.unipark.com) automatically excludes email addresses from further mailings when the receiving server rejects the email or does not respond at all. Other reasons for undeliverable emails such as long absence of the addressee cannot be detected by the tool. We sent a reminder to 64,255 addresses between March, 17^th^ and March, 21^st^. All in all, the survey was completed 1,153 times, a response rate of about 1.8% (see Table 1). While this is obviously a very low response rate, the absolute number of participants still allows for reliable estimates in the statistical analyses. Particularly, the randomization of the participants into the four experimental groups worked, as can be seen by the relatively even distribution among the four groups. Furthermore, we examined the distribution of the citing and cited journals (occurring at least ten times in our survey) for each of the four experimental groups (Table A1 in S1 Appendix). Since there is no clear pattern in this distribution visible, this result is another indicator for a successful randomization.

**Table 1:** Number of participants in four experimental groups.

Experimental group	n	Percent
No information (control)	260	22.5
Access code information	297	25.8
Paper impact information	292	25.3
Journal impact information	304	26.4
Total	1,153	100.0

As not all participants were able to rate the selected reference, ratings from 1,109 participants are available for the analyses. However, too few papers have been rated several times (less than 5% as Table 2 reveals) to conduct fixed effects analyses outlined in our registered protocol [17]. Therefore, we used OLS regression models instead.

**Table 2:** Number of rankings of single papers.

Papers … times ranked	n	Percent
1	1,011	95.8
2	37	3.5
3	4	.4
4	3	.3

Table 3 shows fictitious example data for a cited reference. Cited article 1 received more citations than 40% of the articles from the same subject categories and publication year, and was published in a journal with a higher citation impact than 22% of the journals in the same subject category and year. This information was presented to respondents 1 and 2, respectively. Respondent 3 entered the survey with the access code 80. Respondent 4 assessed the cited reference without indicating an access code in terms of numbers or receiving citation impact percentile (on the paper or journal level).

**Table 3:** Fictitious example data that are paper-based (e.g. the citation impact percentile) or respondent-based (e.g. the quality assessment).

	Paper impact percentile	Journal impact percentile	Access code percentile	No information
**Data of four papers**	1	2	3	4
Percentile	40	22	80	
**Survey data from four respondents**	1	2	3	4
Quality	35	25	45	20
Novelty	30	45	22	35
Significance	21	32	78	74
Validity	34	47	57	76
Generalizability	44	55	83	42
Canonical reference	20	12	15	95

### Regression models

The web-based survey yielded quality assessments of cited articles (concerning quality, novelty, significance, validity, generalizability, and canonical or prominent reference) on a percentile scale from 1 (very poor) to 100 (exceptional) whereby 50 denotes a typical paper. These quality assessments are the dependent variable in the regression models, and we were interested in how they relate to the independent variables: paper impact information, journal impact information, and access code (in terms of percentile values).

The model is specified as follows:

$$quality\;assessment_{j}=\beta_{0}+\beta_{1}condition2+\beta_{2}condition3+\beta_{3}condition1∙percentile+\beta_{4}condition2\cdot percentile+\beta_{5}condition3\cdot percentile+e_{j},$$

whereby *quality assessment*_*j*_ is the quality assessment for the cited article *j* by the respondent, *condition1* (access code), *condition2* (journal impact information), and *condition3* (paper impact information) are dummy variables indicating the respective conditions (treatment groups), *percentile* is the percentile value of access code, journal impact factor or citation impact, and *e*_*j*_ is an error term. In order to facilitate the interpretation of the results, this model does not include a main effect of the percentile, but three interactions. The coefficients of the interaction terms represent the effects of the percentile value on the quality assessment for the three treatments (access code, journal impact information or paper impact information).

## Results

### Average quality assessments

In the first step of the analyses, we compared average quality assessments of articles whose treatment percentile (access code, paper impact, or journal impact percentile) is at or above the median with articles below the median. Due to skewed distributions of citation and journal impact factor percentiles, values at the median were included [17]. Table 4 shows the arithmetic means for the three treatment groups as well as the arithmetic means for all ratings from treatment and control groups.

**Table 4:** Quality assessments of articles with treatment percentiles below and at/above the median.

Experimental group	Treatment percentile below median Mean (variance)	Treatment percentile at or above median Mean (variance)	Overall Mean (variance)
No information (control)			77.0 (252.6)
Access code information	75.3 (299.2)	76.0 (253.3)	75.7 (275.0)
Paper impact information	75.3* (236.5)	79.0* (210.3)	77.4 (223.8)
Journal impact information	75.5 (280.7)	77.6 (260.7)	76.6 (271.1)
Overall			76.6 (256.0)

Note. * *p* < .05.

The overall arithmetic means for the treatment and control groups are very similar (see the overall column in Table 4). This suggests that the type of information that is shown (or if any information is shown at all) did not influence the ratings. However, differences between the types of information become visible when treatment percentiles below and at or above medians are compared. There seems to be no anchoring effect by the anchor with irrelevant quality information as the difference of the average quality assessments for papers with a low (75.3) or high (76.0) code is very close and the difference is not statistically significant. The journal impact information also does not make a great difference and is not statistically significant. Information on the paper impact (an anchor with relevant quality information), however, does have an effect: whereas papers with citation impact below the median are rated at 75.3 on average, papers at or above the median are rated at 79.0. This difference is statistically significant at the 5% level according to a Mann-Whitney test (*z* = −2.02, *p* = .04).

**Fig 1:**
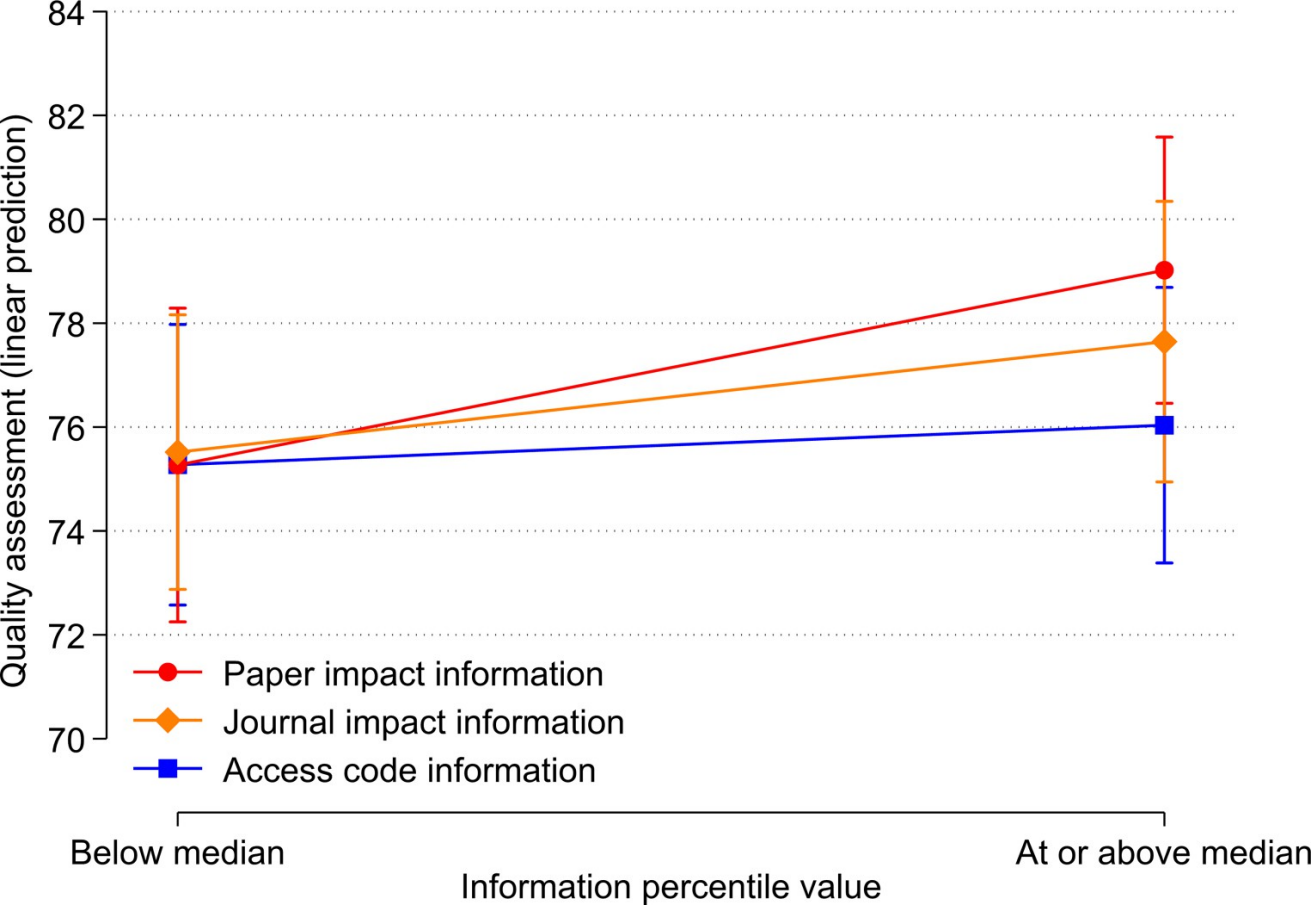
Relationship between treatments (access code, journal impact, and paper impact information) and quality assessment by respondents.

The results suggest that the respondents’ ratings are influenced by the paper impact information, but not the journal impact information or even irrelevant information (the access code). To demonstrate these differences between types of information and treatment percentiles below and above medians, we visualized the results from Table 4 and Fig 1.

### Effect heterogeneity

The finding of no anchoring effect by access code and journal impact is confirmed by the results of the regression analysis in Table 5. The results reveal that there is no effect of the access code percentile on the quality assessment, but a small, yet statistically significant (*p =* .01) effect of the journal impact information and a stronger, also statistically significant (*p* = .03) effect of the paper impact information.

**Table 5:** Regression of quality assessment on information percentile values.

Variable	Coefficient
Paper impact information	-64.60*
(Reference group: Access code information)	(-2.06)
Journal impact information	-8.600
(Reference group: Access code information)	(-1.93)
Access code information x percentile	0.00497
	(0.15)
Paper impact information x percentile	0.682*
	(2.13)
Journal impact information x percentile	0.122*
	(2.47)
Constant	75.41***
	(40.38)
*N*	809
*R* ^2^	0.015

Notes. *t* statistics in parentheses

* *p* < .05

*** *p* < .001.

**Fig 2:**
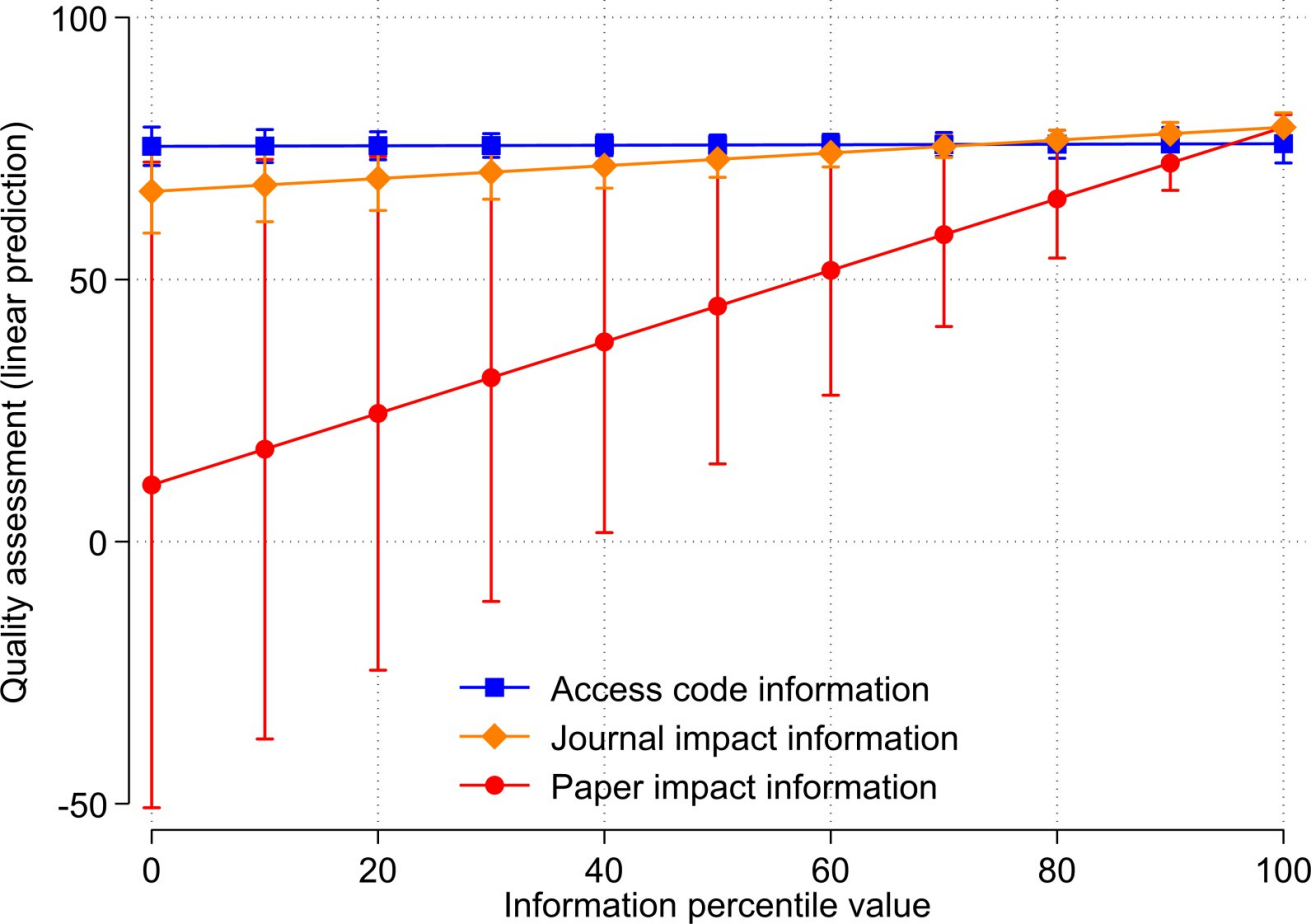
Relationship between treatments (access code, journal impact information, and paper impact information) and quality assessment by respondents.

Fig 2 shows the predicted quality assessments (which resulted from the regression analysis) for access code, paper impact information and journal impact information in terms of percentiles in the range [0, 100]. For treatment values of 100, the predicted quality assessments barely differ between the treatment groups. In contrast, the predicted assessments for low paper impact percentiles are substantially smaller than for low journal impact percentiles or access code percentiles. In the interpretation of the results in Fig 2 it should be considered that all assessed references by the respondents have very high citation impact percentiles and high journal impact percentiles. Therefore, all estimates in the figure are based on (the linear trend among) references that have high citation and journal impact percentiles. Even though predicted assessments for small treatment values are visible in Fig 2, they are not based on actual assessed references with small citation or journal impact percentiles. The main effects of the experimental groups in Table 5 should not be interpreted as they reflect differences in the quality assessments when the respective percentile value is zero.

A very similar picture as in Fig 2 can be observed for all quality related assessments by the respondents (e.g. novelty and significance; see Fig A1 and Table A2 in S1 Appendix). This could be expected, as all assessments are positively correlated with the overall quality assessment and with each other (see Table 6 with the correlation coefficients).

**Table 6:** Correlations (Sperman’s rho coefficients) between quality related assessments and (overall) quality assessment.

Assessment	Overall quality	Novelty	Significance	Validity	Generalizability
Novelty	0.548***				
Significance	0.614***	0.545***			
Validity	0.603***	0.417***	0.564***		
Generalizability	0.420***	0.266***	0.440***	0.485***	
Canonical reference	0.461***	0.284***	0.558***	0.453***	0.423***
Prominent reference	0.577***	0.506***	0.637***	0.564***	0.487***

Note. *** *p* < .001.

Our main result thus far is a statistically significant effect of paper impact percentiles on quality assessments by respondents (who received this information). This result is, however, difficult to interpret. There are two possible interpretations: (1) the respondents adjusted their quality assessments towards the given paper impact information (anchor effect hypothesis). (2) The respondents’ quality assessments are related to the paper impact information, because both are able to reflect the intrinsic quality of single papers (quality effect hypothesis). To find hints towards one of both possible interpretations, we tested additionally whether the same effect as in Fig 2 is also visible in the group of respondents who did not receive the information on paper impact (i.e. respondents in the control group). The same result would have meant that the anchor effect hypothesis is wrong.

**Fig 3:**
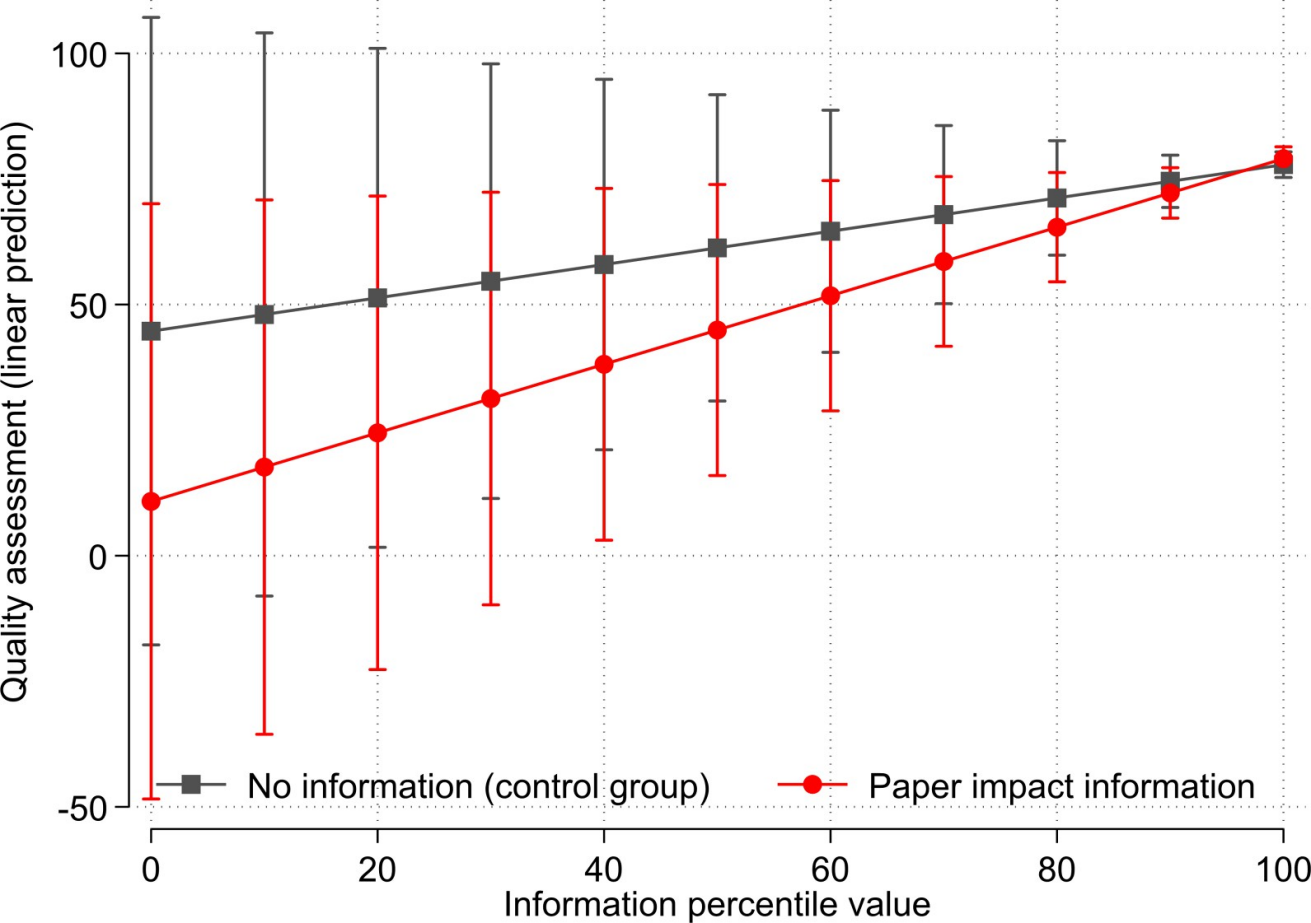
Relationship between paper impact percentile and quality assessment for two groups: (1) respondent received information about paper impact percentile, and (2) respondent did not receive information about paper impact percentile.

The results for the test of the two possible interpretations are presented in Fig 3 and Table A3 in S1 Appendix. Although the results for the control group (respondents who did not receive citation impact information) reflect a degree of relationship between paper impact and quality assessment, the anchor seemed to work for the respondents with impact information. The effect of the paper impact information on the quality assessment is clearly visible: the presentation of the paper impact information appears to decrease the quality assessments by the respondents more strongly compared to the control group. The effect of the paper impact information is statistically significant in the group which received this information while it is not in the control group. The results, therefore, appear to tend more in the direction of the validity of the anchor effect hypothesis.

### Robustness checks

#### Presentation of questions in different order

**Fig 4:**
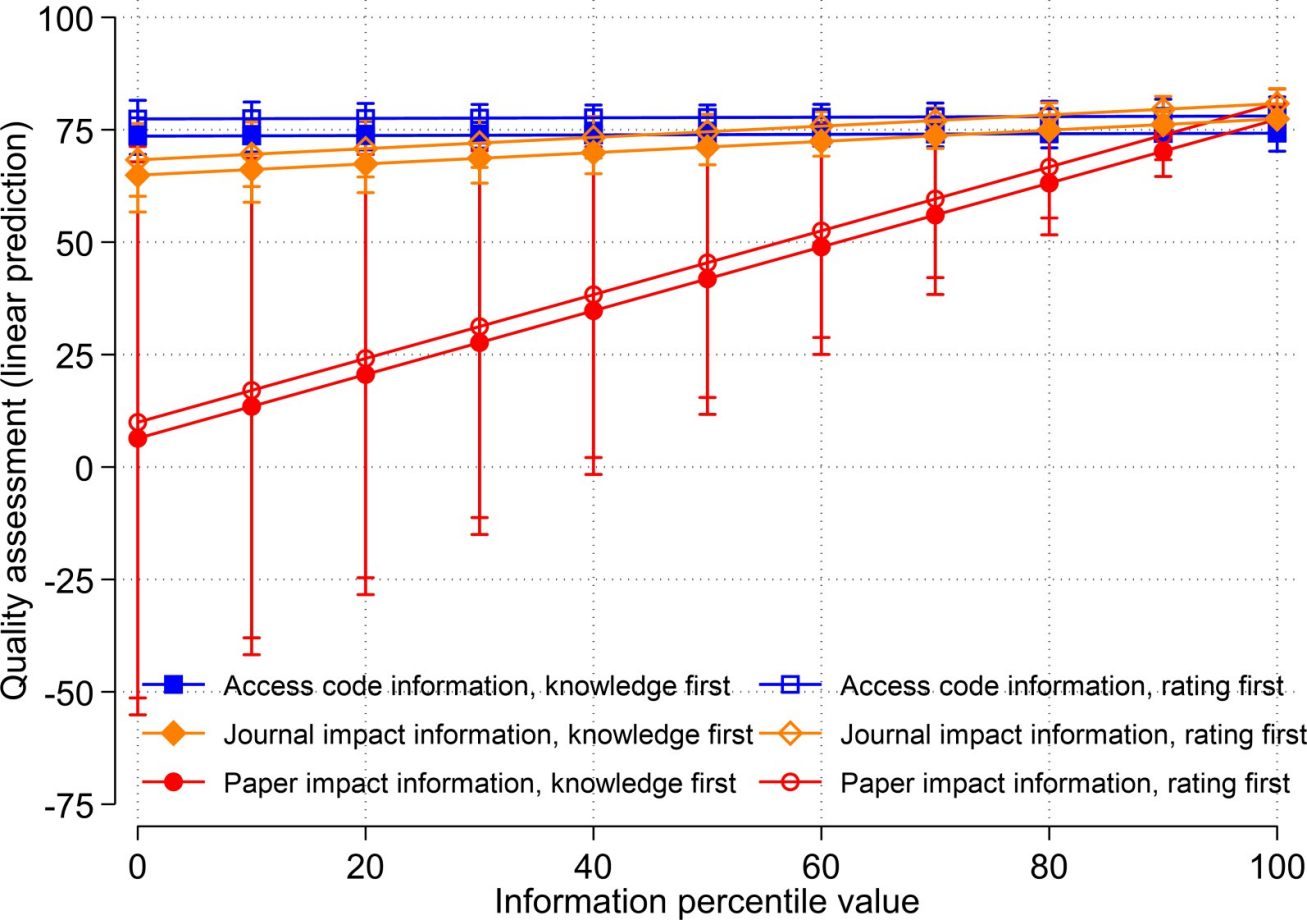
Relationship between treatments (access code information, journal impact information, and paper impact information) and quality assessments depending on question order (knowledge or rating is presented first).

In the survey, the order of the questions regarding the references was randomized. 52.2% of the respondents first answered the questions how well they remembered the cited paper, how much the reference influenced their research choices in the citing paper (and which choices), and were then confronted with the questions concerning the quality of the cited paper. The other 47.5% answered the questions the other way around. A regression model including interaction terms of the treatment group dummies and a dummy representing the question order shows no substantial differences to the model reported in the previous section (see Fig 4, Table A4 in S1 Appendix). In other words, we also found no effect of the access code percentile whereas the paper impact and journal impact percentiles have a statistically significant effect on the quality assessment.

#### Prior knowledge of our study

**Fig 5:**
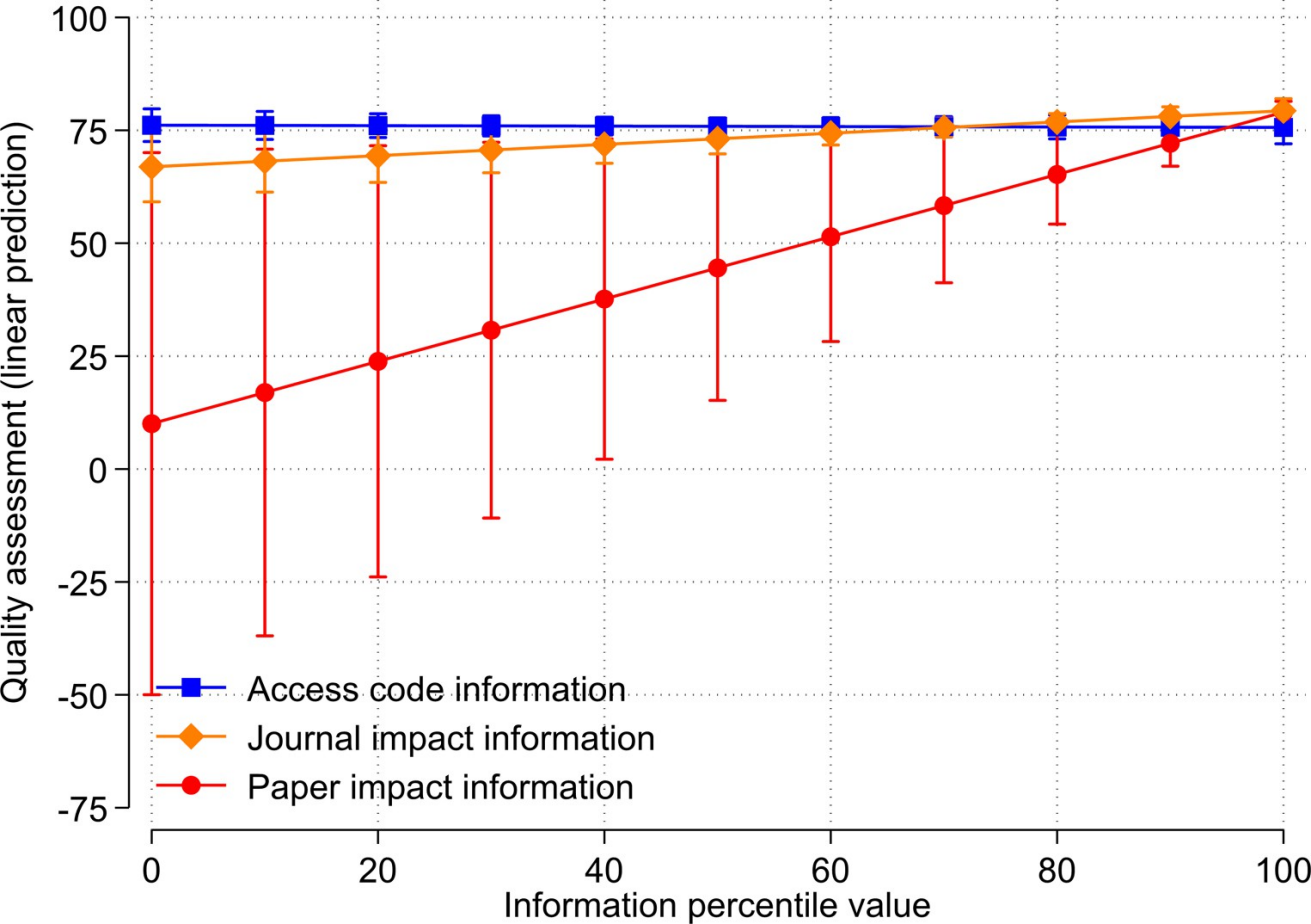
Relationship between treatments (access code information, journal impact information, and paper impact information) and quality assessments. Eighteen respondents with prior knowledge on our survey and/or Teplitksy, Duede (1) are excluded.

Knowledge of our research goals might have biased the respondents’ answers. As the results show, however, our expectations were not confirmed: when we excluded the 18 respondents who had read our registered report protocol [17] or the replicated study [10], our results remain virtually unchanged (Fig 5, Table A4 in S1 Appendix).

#### Knowledge of the cited reference

**Fig 6:**
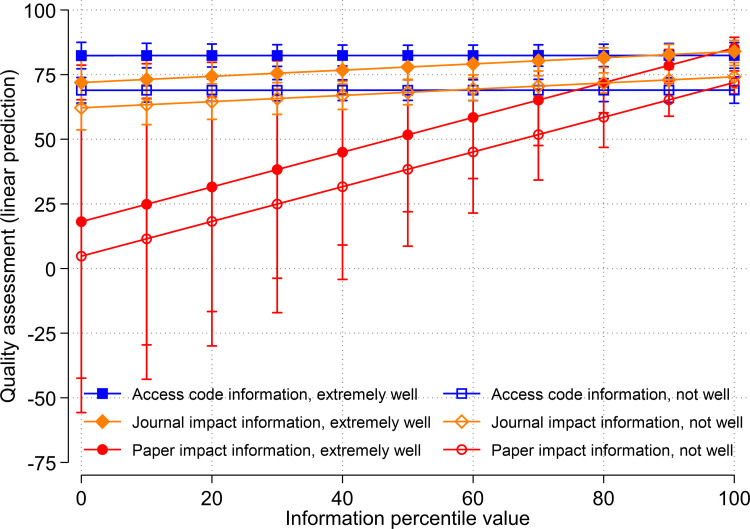
Relationship between treatments (access code information, journal impact information, and paper impact information) and quality assessments for respondents with extremely good knowledge (solid symbols) and poor knowledge (hollow symbols) of the cited paper.

In the final robustness check, we included interaction terms of treatment group dummies and a dummy indicating whether the respondents knew the cited paper which was rated well or not. For not well-known articles, the respondents may be more likely to base their assessments on additional information (i.e. the presented anchor values). If this is the case, anchoring effects should be stronger for cited papers that are not well-known to the respondents. The results in Fig 6 and Table A4 in S1 Appendix show that less knowledge of the cited paper leads to lower rankings. We suspect that the citing authors had read more thoroughly those papers which they assessed as good papers in our survey, and the other way around. Despite the deviations, our main finding remains unchanged as Fig 6 demonstrates. The results reveal that an anchor effect of paper impact information exists.

## Discussion

Replicating the study design of Teplitskiy, Duede [10], we conducted a survey to study assessments of cited papers within the framework of the anchoring-and-adjustment heuristic. Our results mainly confirm the results by Teplitskiy, Duede [10]: the quality assessments of papers seem to depend on the citation impact information of single papers. The other information (anchors), however, such as an arbitrary number (an access code) and journal impact information did not play a (important) role in the assessments of papers. In our survey, we experimentally manipulated the information citing authors (respondents) received when assessing papers that they have cited. The control and treatment forms were mainly identical. However, each treatment group received varying anchor information: two groups received the true paper impact and journal impact information and one group received a random number without any relation to quality.

On the one hand, the missing effect of the arbitrary number in our study does not agree with the results of previous studies. Furnham and Boo [4] summarized the literature and found that “irrelevant anchors produce similar effects in judgmental decisions in comparison to those of informational relevance anchors” (p. 38). Similar conclusions can be found in Mussweiler, Englich [11]. On the other hand, a small effect of the journal impact information as we found has been reported in previous studies. Carpenter, Sarli [25] investigated the relationship of the ‘BEEM’ (best evidence in emergency medicine) rater scale, journal impact factor, and citation counts. Their results are as follows: “The performance of the BEEM rater score was assessed for each article using negative binomial regression with composite citation count as the criterion standard, while controlling for other independent bibliometric variables in three models …The citation rate and BEEM rater score correlated positively (0.144), while the BEEM rater score and the Journal Citation Report (JCR) impact factor score were minimally correlated (0.053)”. It seems, therefore, that the journal impact information influences the assessment of papers but not strongly.

In contrast to the journal impact and access code information, we found a considerable effect of the paper impact information on the quality assessments of papers. However, it is not completely clear how to interpret the result. Since the paper impact information is information which is related to quality, it is possible that the respondents either used the information to assess the cited reference or the information reflects the assessments by the respondents (that they had also done without the information). To receive hints for a conclusive interpretation of our results, we compared quality assessments of papers of two groups: one group received the paper impact information in the survey; the other group did not receive the information (or any other information), but we had the information in our dataset. Thus, we could compare how paper impact information is related to quality assessments for respondents who received the information or not.

Since our results point out that the quality assessments were assimilated toward the paper impact information, there seems to be an anchoring bias caused by insufficient adjustment. This result is in line with one of the main findings by Teplitskiy, Duede [10]. It seems that the respondents assessed cited papers worse when they observed rather low paper impact values in the survey. The use of citation counts as an anchor may follow the mechanism proposed by Mussweiler, Englich [11]: “a particular value may be selected as an anchor because conversational inferences suggest it as relevant. If a particular anchor is explicitly mentioned by the experimenter, then judges may well use it to subsequently compare it to the target” (p. 195).

The missing effect of the access code may be interpreted as a good sign for quality assessments of papers: it seems that these assessments cannot be influenced by irrelevant information. The slight effect of the journal impact information may also be seen as a good sign: many initiatives such as DORA (see https://sfdora.org) and the Leiden manifest [26] exist with the goal of reducing the use of journal impact information in research evaluation processes. With the use of these information, there is the danger that a single paper is assessed with an average value across all papers in a journal. Since citation data usually follow a skewed distribution, the average value is a bad representative of the citation impact of single papers. Our results with respect to the journal impact information can be interpreted in two ways: (1) the initiatives may have led to the desired goal: journal impact information plays no longer an important role in paper assessments. (2) The initiatives overestimated the influence of the information: scientists do not use the information as extensively as feared.

Although the comparison of the various groups in our study may be interpreted as a confirmation of the paper impact information as anchor, the design of our analysis does not allow this strong causal conclusion. The results are based on a limited design, since the respondents in the groups (receiving various information or not) did not assess the same cited paper: the cited paper was not held constant in the assessment by respondents with or without the information. Although we used an experimental design in our study, we cannot rule out completely the possibility that the assessment of different papers (with different quality) leads to the observed quality assessments. We propose therefore to study bibliometrics within the anchoring-and-adjustment heuristic with an optimized design in a future study. We initially planned for our study [17] that the same cited reference is multiply assessed by respondents who are randomly assigned to different treatment groups. Since we did not receive enough completed questionnaires for single papers, we could not analyze the data accordingly.

We also recommend for the optimized design that the research fields of the respondents are controlled in the statistical analyses. The top cited journals in Table A1 in S1 Appendix have a general life science focus. Since bibliometrics have an important role in life sciences [1], the statistically significant effect of the paper impact information might result from this focus (partly). Another field-specific focus of our study might have resulted in different results.

## Supporting information

S1 Appendix (DOCX)
