# Supplementary material for: Anchoring effects in the assessment of papers: An empirical survey of citing authors
Source: PLoS One. 2023 Mar 31;18(3):e0283893. doi: 10.1371/journal.pone.0283893 (PMC10065272; doi:10.1371/journal.pone.0283893)
Supplement: S1 Appendix — (DOCX) [file pone.0283893.s001.docx]

# Appendix

Table A1: Distribution of respondents on experimental groups by citing and cited journals

| Journal | No information (control group) | Access code information | Paper impact information | Journal impact information | Total |  |
| --- | --- | --- | --- | --- | --- | --- |
| Top citing journals | | | | | | |
| *PLOS ONE* | 3 | 4 | 5 | 4 | 16 |  |
| *Scientific Reports* | 5 | 2 | 3 | 6 | 16 |  |
| Total | 8 | 6 | 8 | 10 | 32 |  |
| Top cited journals | | | | | | |
| *Annals of Internal Medicine* | 2 | 2 | 5 | 1 | 10 |  |
| *Journal of Clinical Oncology* | 2 | 3 | 2 | 5 | 12 |  |
| *Nature* | 5 | 5 | 8 | 9 | 27 |  |
| *New England Journal of Medicine* | 5 | 3 | 6 | 4 | 18 |  |
| *Pediatrics* | 2 | 2 | 2 | 4 | 10 |  |
| *Physical Review Letters* | 6 | 5 | 5 | 2 | 18 |  |
| *Science* | 4 | 7 | 5 | 2 | 18 |  |
| *The Lancet* | 4 | 5 | 8 | 6 | 23 |  |
| Total | 30 | 32 | 41 | 33 | 136 |  |

Figure A1. Relationship between treatments (access code information, journal impact information, and paper impact information) and assessments by respondents with respect to novelty (departed significantly from what is usually done in this research area), significance (addressed an important topic), validity (designed and performed very well, produced very credible results), generalizability (easily applicable to other contexts), canonical reference (the standard reference for this topic), and prominent reference (widely known in the scientific community).

Table A2. Regression models of quality aspects on information percentile values

| Variable | (1) Novelty | (2) Significance | (3) Validity | (4) Generalizability | (5) Canonical | (6) Prominent |
| --- | --- | --- | --- | --- | --- | --- |
|  | Coefficient | Coefficient | Coefficient | Coefficient | Coefficient | Coefficient |
| Paper impact information | -60.51 | -66.35^*^ | -70.47^*^ | -76.52^*^ | -48.68 | -130.1^*^ |
| (Reference group: Access code information) | (-1.58) | (-2.22) | (-2.02) | (-2.13) | (-1.00) | (-2.33) |
|  |  |  |  |  |  |  |
| Journal impact information | -8.417 | -7.061 | -10.81^*^ | -3.270 | -0.437 | -5.299 |
| (Reference group: Access code information) | (-1.52) | (-1.47) | (-2.18) | (-0.55) | (-0.05) | (-0.60) |
|  |  |  |  |  |  |  |
| Access code information x percentile | -0.00282 | 0.0428 | -0.00294 | 0.00454 | 0.0718 | 0.0342 |
|  | (-0.07) | (1.25) | (-0.08) | (0.11) | (1.17) | (0.59) |
|  |  |  |  |  |  |  |
| Paper impact information x percentile | 0.622 | 0.706^*^ | 0.707^*^ | 0.829^*^ | 0.551 | 1.439^*^ |
|  | (1.59) | (2.31) | (1.99) | (2.25) | (1.10) | (2.53) |
|  |  |  |  |  |  |  |
| Journal impact information x percentile | 0.119 | 0.133^*^ | 0.118^*^ | 0.0405 | 0.0534 | 0.183 |
|  | (1.94) | (2.51) | (2.15) | (0.61) | (0.56) | (1.85) |
|  |  |  |  |  |  |  |
| Constant | 70.17^***^ | 75.37^***^ | 79.53^***^ | 71.03^***^ | 72.16^***^ | 69.51^***^ |
|  | (30.38) | (37.72) | (38.08) | (29.17) | (20.79) | (20.06) |
| *N* | 774 | 825 | 783 | 786 | 390 | 396 |
| *R*^2^ | 0.009 | 0.017 | 0.012 | 0.016 | 0.009 | 0.059 |

Notes. Novelty (departed significantly from what is usually done in this research area), significance (addressed an important topic), validity (designed and performed very well, produced very credible results), generalizability (easily applicable to other contexts), canonical reference (the standard reference for this topic), and prominent reference (widely known in the scientific community).

*t* statistics in parentheses; **p*<.05, ****p*<.001

Table A3. Regression model of quality assessments on paper impact information and group membership

| Variable | Coefficient |
| --- | --- |
| Paper impact information | -33.89 |
| [Reference group: No information (control group)] | (-0.77) |
|  |  |
| No information (control group) x percentile | 0.331 |
|  | (1.02) |
|  |  |
| Paper impact information x percentile | 0.682^*^ |
|  | (2.21) |
|  |  |
| Constant | 44.70 |
|  | (1.41) |
| *N* | 487 |
| *R*^2^ | 0.012 |

Notes. *t* statistics in parentheses; * *p*<0.05

Table A4. Regression models of quality assessments on information percentile values.
Model 1: interactions with question order, model 2: respondents with prior information on our survey excluded, model 3: interactions with knowledge of cited papers included.

| Variable | (1) Question order | (2) Respondents excluded | (3) Knowledge |
| --- | --- | --- | --- |
|  | Coefficient | Coefficient | Coefficient |
| Paper impact information | -67.21^*^ | -66.09^*^ | -64.25^*^ |
| (Reference group: Access code information) | (-2.14) | (-2.16) | (-2.07) |
|  |  |  |  |
| Journal impact information | -8.678 | -9.220^*^ | -11.33 |
| (Reference group: Access code information) | (-1.86) | (-2.12) | (-1.96) |
|  |  |  |  |
| Access code information x percentile | 0.00661 | -0.00487 | 0.000617 |
|  | (0.21) | (-0.15) | (0.02) |
|  |  |  |  |
| Paper impact information x percentile | 0.710^*^ | 0.690^*^ | 0.672^*^ |
|  | (2.22) | (2.20) | (2.13) |
|  |  |  |  |
| Journal impact information x percentile | 0.125^*^ | 0.124^*^ | 0.120^*^ |
|  | (2.54) | (2.58) | (2.47) |
|  |  |  |  |
| Access code information x rating first | 3.813^*^ |  |  |
|  | (1.98) |  |  |
|  |  |  |  |
| Paper impact information x rating first | 3.579 |  |  |
|  | (1.81) |  |  |
|  |  |  |  |
| Journal impact information x rating first | 3.395 |  |  |
|  | (1.77) |  |  |
|  |  |  |  |
| Access code information x knowledge of cited paper |  |  | -3.354^***^ |
|  |  |  | (-3.76) |
|  |  |  |  |
| Paper impact information x knowledge of cited paper |  |  | -3.336^***^ |
|  |  |  | (-3.68) |
|  |  |  |  |
| Journal impact information x knowledge of cited paper |  |  | -2.446^**^ |
|  |  |  | (-2.83) |
|  |  |  |  |
| Constant | 73.59^***^ | 76.13^***^ | 85.72^***^ |
|  | (35.45) | (41.29) | (25.99) |
| *N* | 809 | 791 | 806 |
| *R*^2^ | 0.028 | 0.016 | 0.057 |

Notes. *t* statistics in parentheses; * *p*<0.05, ** *p*<0.01, *** *p*<0.001
